# Announcement: ESF Workshop on "Molecular Recognition in Metalloproteins"

**DOI:** 10.1155/MBD.1997.116

**Published:** 1997

**Authors:** M. A. De la Rosa

**Affiliations:** Instituto de Bioquimica Vegetal y Fotosintesis Centro Isla de la Cartuja Universidad de Sevilla y CSIC Sevilla Americo Vespucio 41092 Spain


					ESF WORKSHOP ON "MOLECULAR RECOGNITION IN
METALLOPROTEINS"
23- 26 October 1997
Sevilla, Spain
Organized by Miguel A. De la Rosa (Sevilla) & Carlos Gomez-Moreno (Zaragoza)
SCOPE AND AIM
One of the main goals of modern protein chemistry is the understanding of structure-function
relationships in biological molecules, that is, how a molecule with a precise spatial conformation and
specific properties plays a particular role in the gearing of the cellular machinery. This is what we
now call "molecular recognition", the means by which molecules recognize other molecules to
interact with them either forming active complexes so as to fulfill a specific role, such as
transferring atoms or electrons between them, or for any other function as well. Despite the fact
that we know a great deal about molecular recognition, even more still remains unknown: How
molecules interact one with others inside the cells? What are the factors that control the reaction
mechanisms? Which are the structural features that determine the physiological function? During
this three-day meeting in Seville the state-of-the-art on this subject will be discussed.
TOPICS TO BE COVERED
Cytochromes and their redox partners
Cu-containing proteins
Ferredoxins and oxidoreductases
Non-redox metalloproteins
Crystal and solution structures of proteins complexes
Algorithms used for prediction of molecular interactions
Current trends in macromolecular recognition
Names of invited speakers (who have already accepted):
X. Aviles (Spain), L. Banci (Italy), R. Bernhardt (Germany), H. Bohme (Germany), H. R. Bosshard
(Switzerland), M. A. Carrondo (Portugal), S. Chapman (UK), R. Crichton (Belgium), J. Fontecilla-
Camps (France), M. Frey (France), J. Hadju (Sweden), M. Hervas (Sevilla), P. Kroneck (Germany), L.
Kuhn (Germany), P. Lindley (UK), M. Medina (Spain), J. Meyer (France), J. Moura (Portugal), I.
Pecht (Israel), S. Sligar (USA), M. Sternberg (UK), G. Sykes (UK), J. Ulstrup (Denmark), G. Zanetti
(Italy).
The opening lecture will be addressed by Prof. R. Huber (Germany), awarded the Nobel Prize in
Chemistry 1988 for the determination of the 3D structure of a photosynthetic reaction center.
Participation is limited to 50 including 25 invited speakers. The fee for attending the meeting
includes accomodation and meals; it amounts to 1,400 FF in double room and 1,600 FF in single
room.
The workshop will be held in Seville, the capital of Andalusia which is an extensive region in South
Spain stretching over the Sierra to the sea. Seville is readily accessible by air, train and road.
Applications, which should include a short CV (1-2 pages), a list of publications, fax number and e-
mail address, should be sent to:
M. A. De la Rosa,
Instituto de Bioquimica Vegetal y Fotosintesis,
Centro Isla de la Cartuja,
Universidad de Sevilla y CSIC,
Americo Vespucio, s/n
41092-Sevilla, Spain
fax: +3454460065
e-mail: Marosa@cica.es)
116

				

